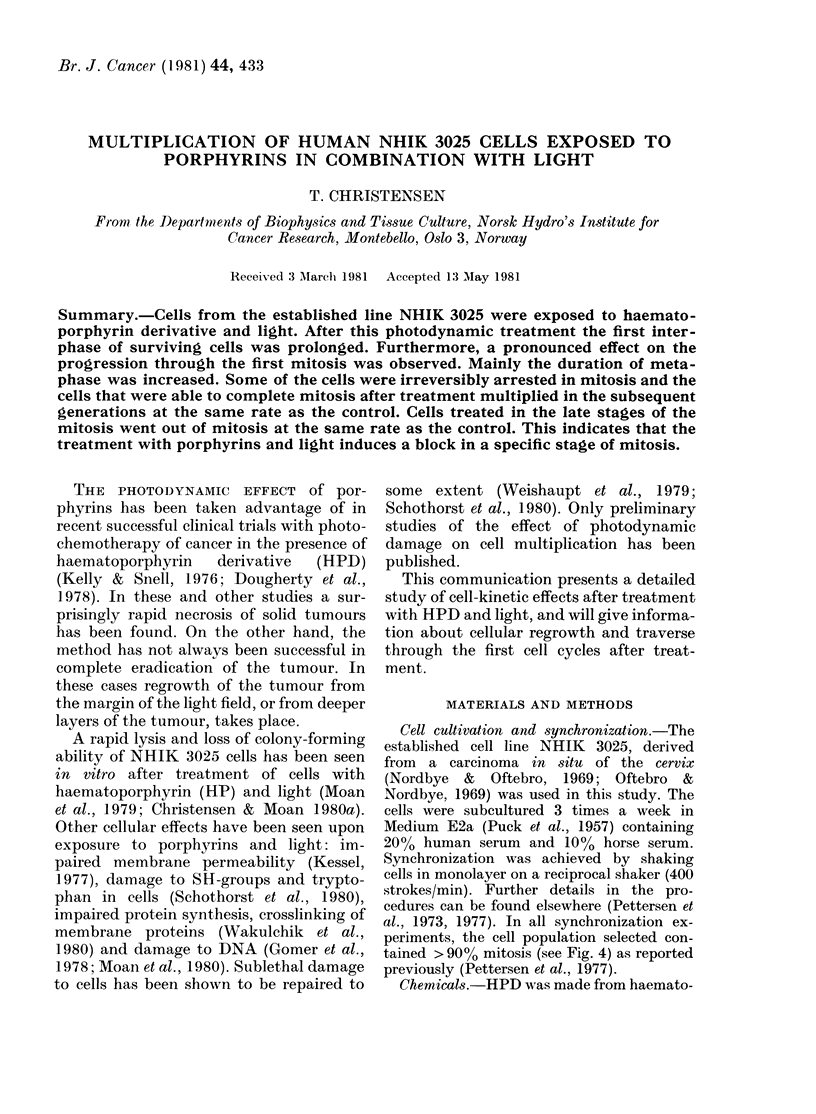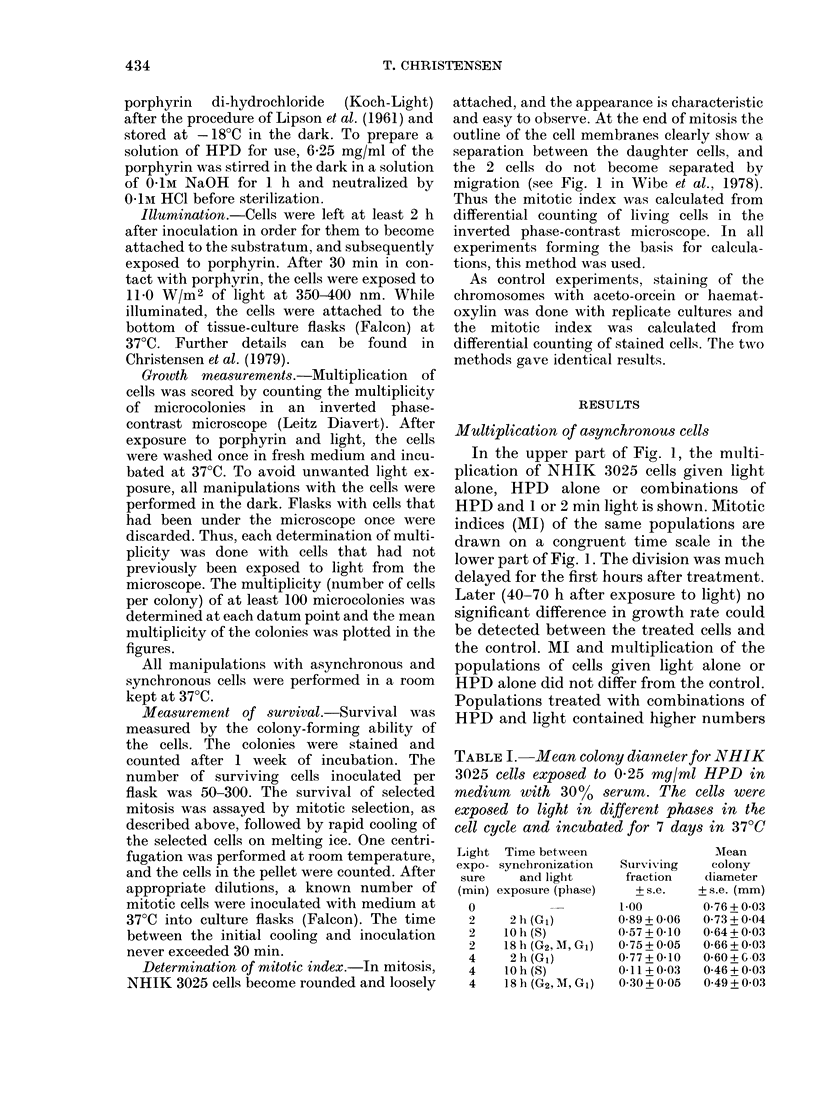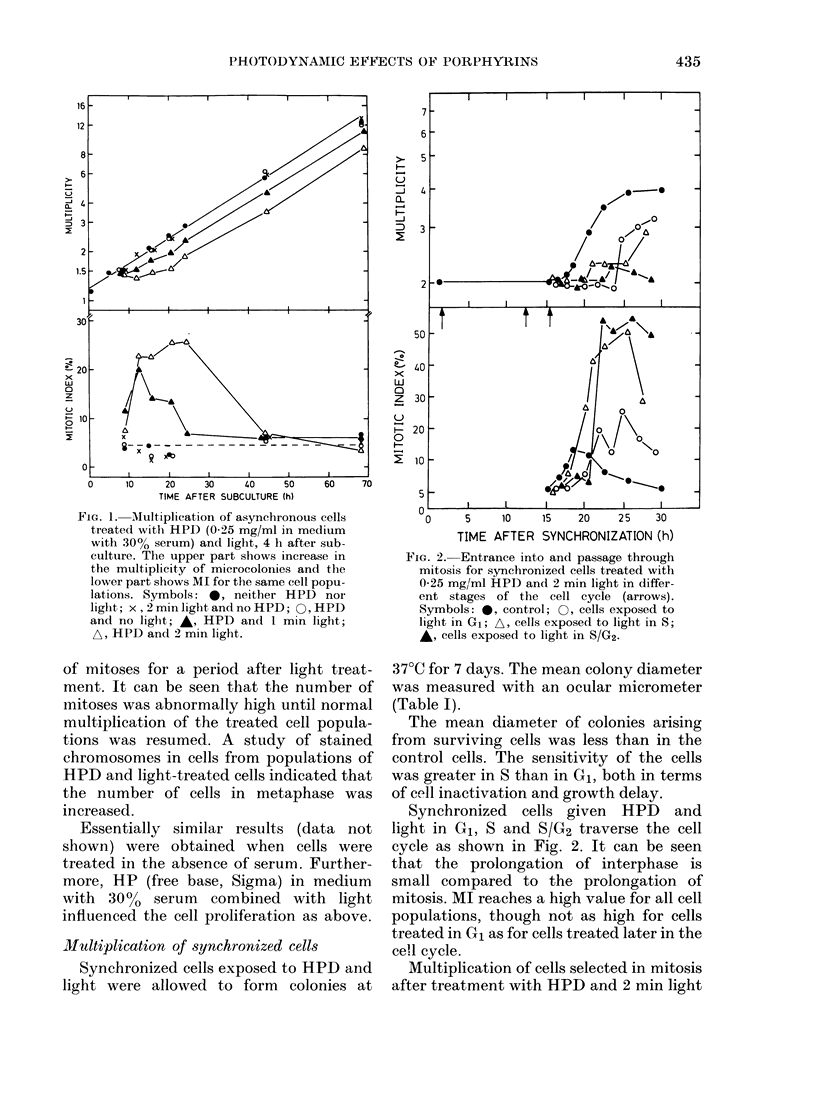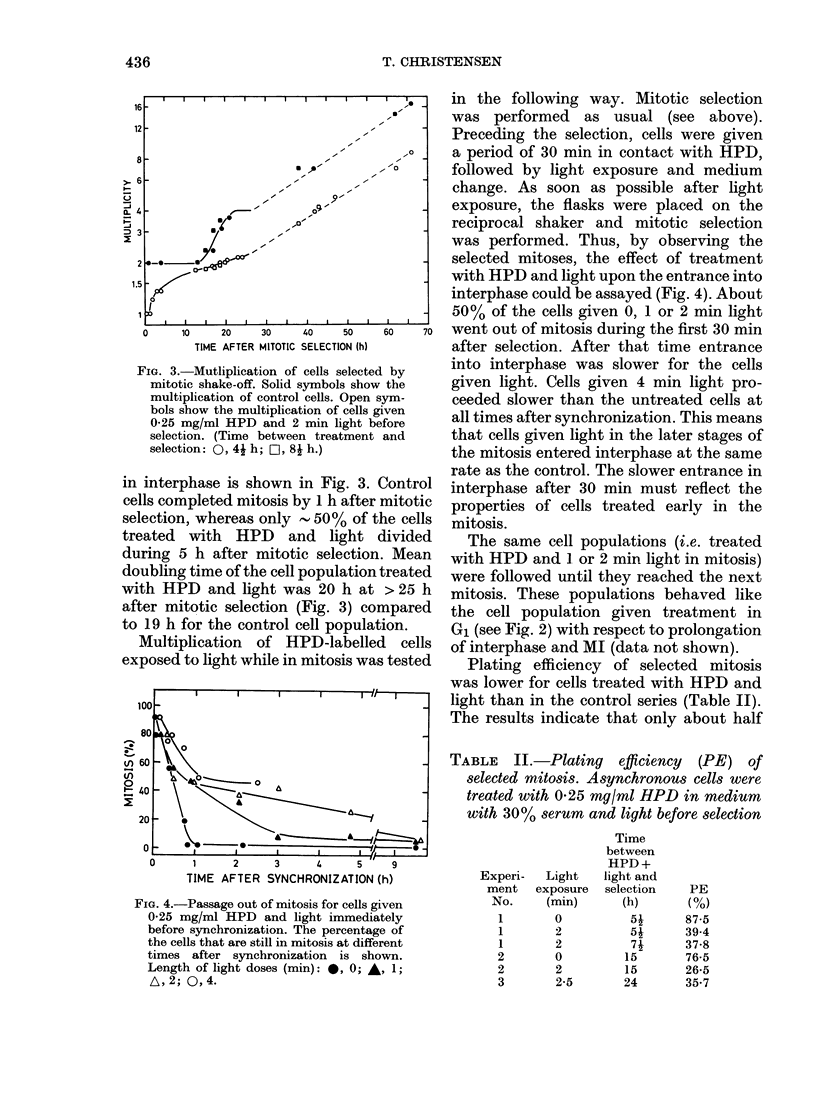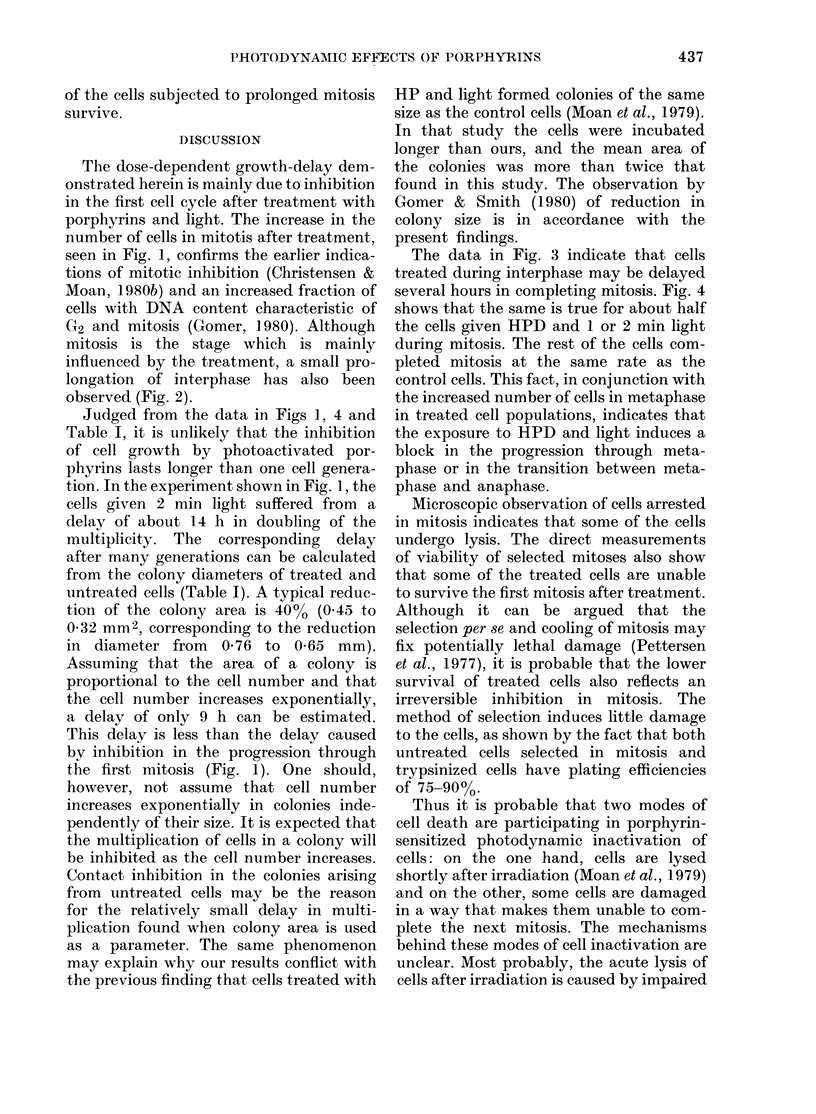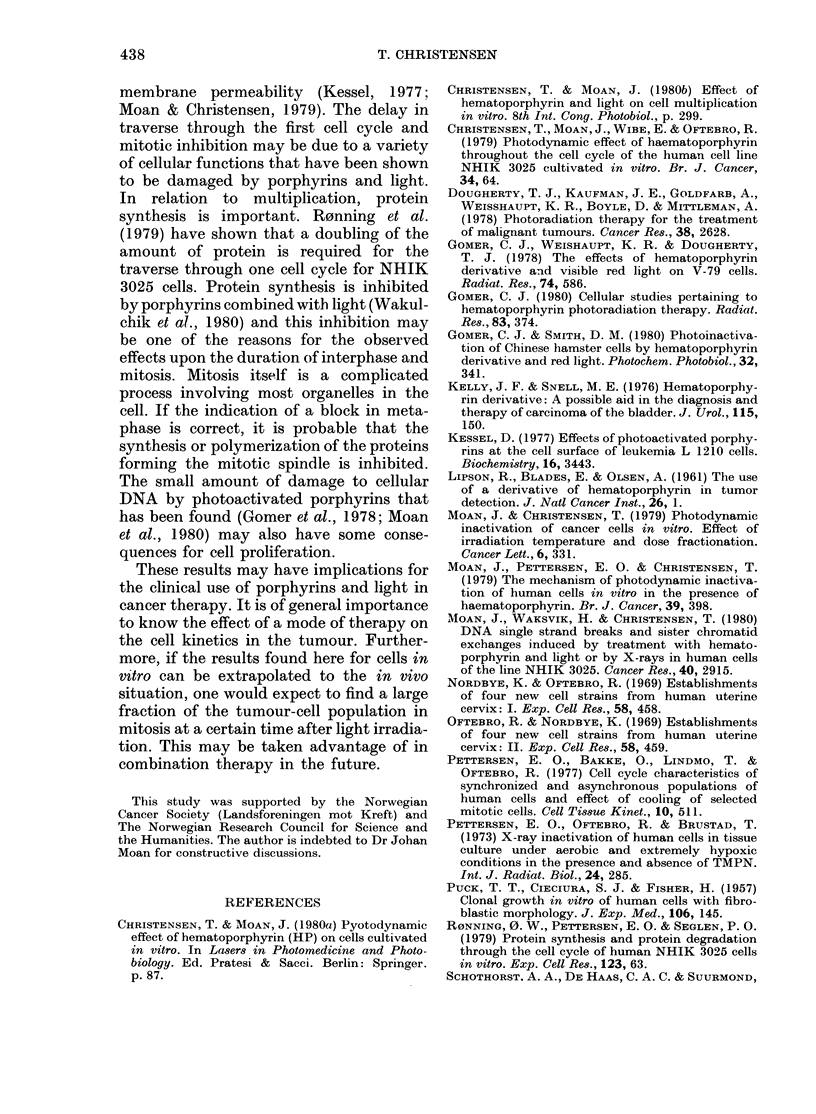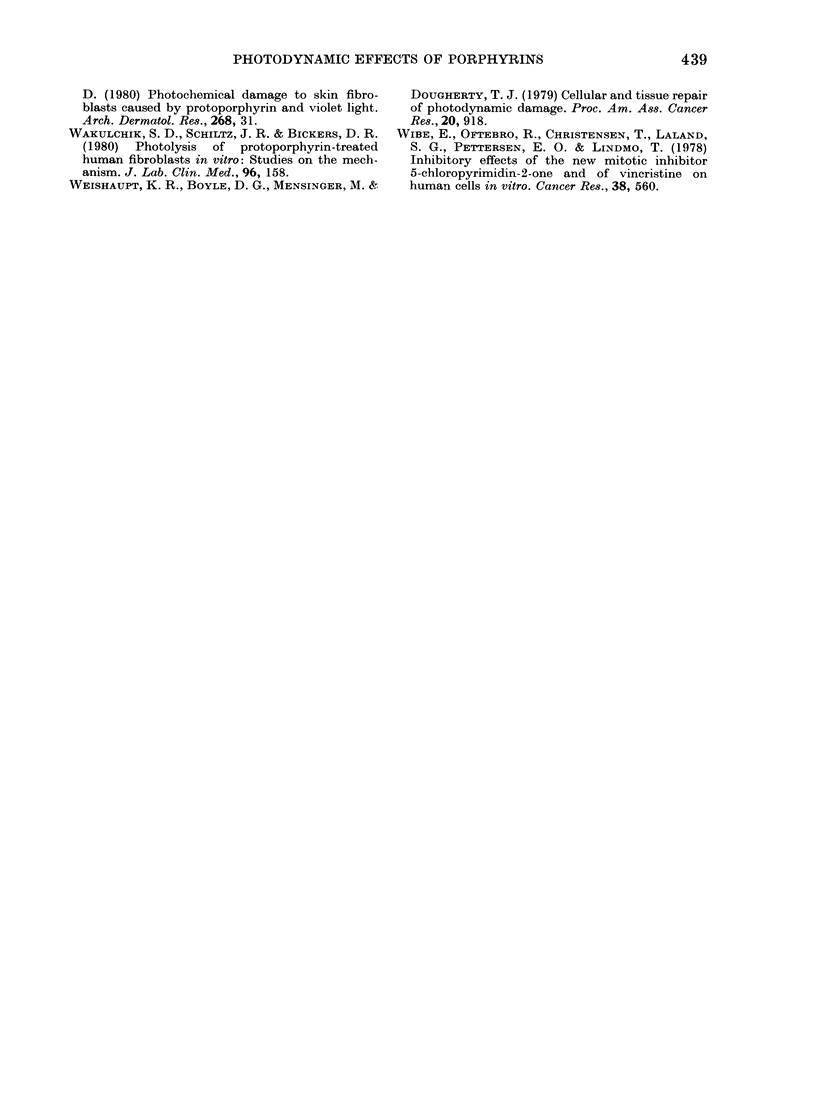# Multiplication of human NHIK 3025 cells exposed to porphyrins in combination with light.

**DOI:** 10.1038/bjc.1981.202

**Published:** 1981-09

**Authors:** T. Christensen

## Abstract

Cells from the established line NHIK 3025 were exposed to haematoporphyrin derivative and light. After this photodynamic treatment the first interphase of surviving cells was prolonged. Furthermore, a pronounced effect on the progression through the first mitosis was observed. Mainly the duration of metaphase was increased. Some of the cells were irreversibly arrested in mitosis and the cells that were able to complete mitosis after treatment multiplied in the subsequent generations at the same rate as the control. Cells treated in the late stages of the mitosis went out of mitosis at the same rate as the control. This indicates that the treatment with porphyrins and light induces a block in a specific stage of mitosis.


*Br. J. Cancer* (1981) **44**, 433

### MULTIPLICATION OF HUMAN NHIK 3025 CELLS EXPOSED TO PORPHYRINS IN COMBINATION WITH LIGHT

T. CHRISTENSEN

*From the Departments of Biophysics and Tissue Culture, Norsk Hydro's Institute for Cancer Research, Montebello, Oslo 3, Norway*

Received 3 March 1981 Accepted 13 May 1981

**Summary.—Cells from the established line NHIK 3025 were exposed to haematoporphyrin derivative and light. After this photodynamic treatment the first interphase of surviving cells was prolonged. Furthermore, a pronounced effect on the progression through the first mitosis was observed. Mainly the duration of metaphase was increased. Some of the cells were irreversibly arrested in mitosis and the cells that were able to complete mitosis after treatment multiplied in the subsequent generations at the same rate as the control. Cells treated in the late stages of the mitosis went out of mitosis at the same rate as the control. This indicates that the treatment with porphyrins and light induces a block in a specific stage of mitosis.**

THE PHOTODYNAMIC EFFECT of porphyrins has been taken advantage of in recent successful clinical trials with photochemotherapy of cancer in the presence of haematoporphyrin derivative (HPD) (Kelly & Snell, 1976; Dougherty *et al.*, 1978). In these and other studies a surprisingly rapid necrosis of solid tumours has been found. On the other hand, the method has not always been successful in complete eradication of the tumour. In these cases regrowth of the tumour from the margin of the light field, or from deeper layers of the tumour, takes place.

A rapid lysis and loss of colony-forming ability of NHIK 3025 cells has been seen *in vitro* after treatment of cells with haematoporphyrin (HP) and light (Moan *et al.*, 1979; Christensen & Moan 1980*a*). Other cellular effects have been seen upon exposure to porphyrins and light: impaired membrane permeability (Kessel, 1977), damage to SH-groups and tryptophan in cells (Schothorst *et al.*, 1980), impaired protein synthesis, crosslinking of membrane proteins (Wakulchik *et al.*, 1980) and damage to DNA (Gomer *et al.*, 1978; Moan *et al.*, 1980). Sublethal damage to cells has been shown to be repaired to some extent (Weishaupt *et al.*, 1979; Schothorst *et al.*, 1980). Only preliminary studies of the effect of photodynamic damage on cell multiplication has been published.

This communication presents a detailed study of cell-kinetic effects after treatment with HPD and light, and will give information about cellular regrowth and traverse through the first cell cycles after treatment.

### MATERIALS AND METHODS

*Cell cultivation and synchronization.*—The established cell line NHIK 3025, derived from a carcinoma *in situ* of the *cervix* (Nordbye & Oftebro, 1969; Oftebro & Nordbye, 1969) was used in this study. The cells were subcultured 3 times a week in Medium E2a (Puck *et al.*, 1957) containing 20% human serum and 10% horse serum. Synchronization was achieved by shaking cells in monolayer on a reciprocal shaker (400 strokes/min). Further details in the procedures can be found elsewhere (Pettersen *et al.*, 1973, 1977). In all synchronization experiments, the cell population selected contained >90% mitosis (see Fig. 4) as reported previously (Pettersen *et al.*, 1977).

*Chemicals.*—HPD was made from haematoporphyrin di-hydrochloride (Koch-Light) after the procedure of Lipson *et al.* (1961) and stored at −18°C in the dark. To prepare a solution of HPD for use, 6·25 mg/ml of the porphyrin was stirred in the dark in a solution of 0·1M NaOH for 1 h and neutralized by 0·1M HCl before sterilization.

*Illumination.*—Cells were left at least 2 h after inoculation in order for them to become attached to the substratum, and subsequently exposed to porphyrin. After 30 min in contact with porphyrin, the cells were exposed to 11·0 W/m$^2$ of light at 350–400 nm. While illuminated, the cells were attached to the bottom of tissue-culture flasks (Falcon) at 37°C. Further details can be found in Christensen *et al.* (1979).

*Growth measurements.*—Multiplication of cells was scored by counting the multiplicity of microcolonies in an inverted phase-contrast microscope (Leitz Diavert). After exposure to porphyrin and light, the cells were washed once in fresh medium and incubated at 37°C. To avoid unwanted light exposure, all manipulations with the cells were performed in the dark. Flasks with cells that had been under the microscope once were discarded. Thus, each determination of multiplicity was done with cells that had not previously been exposed to light from the microscope. The multiplicity (number of cells per colony) of at least 100 microcolonies was determined at each datum point and the mean multiplicity of the colonies was plotted in the figures.

All manipulations with asynchronous and synchronous cells were performed in a room kept at 37°C.

*Measurement of survival.*—Survival was measured by the colony-forming ability of the cells. The colonies were stained and counted after 1 week of incubation. The number of surviving cells inoculated per flask was 50–300. The survival of selected mitosis was assayed by mitotic selection, as described above, followed by rapid cooling of the selected cells on melting ice. One centrifugation was performed at room temperature, and the cells in the pellet were counted. After appropriate dilutions, a known number of mitotic cells were inoculated with medium at 37°C into culture flasks (Falcon). The time between the initial cooling and inoculation never exceeded 30 min.

*Determination of mitotic index.*—In mitosis, NHIK 3025 cells become rounded and loosely attached, and the appearance is characteristic and easy to observe. At the end of mitosis the outline of the cell membranes clearly show a separation between the daughter cells, and the 2 cells do not become separated by migration (see Fig. 1 in Wibe *et al.*, 1978). Thus the mitotic index was calculated from differential counting of living cells in the inverted phase-contrast microscope. In all experiments forming the basis for calculations, this method was used.

As control experiments, staining of the chromosomes with aceto-orcein or haematoxylin was done with replicate cultures and the mitotic index was calculated from differential counting of stained cells. The two methods gave identical results.

### RESULTS

### *Multiplication of asynchronous cells*

In the upper part of Fig. 1, the multiplication of NHIK 3025 cells given light alone, HPD alone or combinations of HPD and 1 or 2 min light is shown. Mitotic indices (MI) of the same populations are drawn on a congruent time scale in the lower part of Fig. 1. The division was much delayed for the first hours after treatment. Later (40–70 h after exposure to light) no significant difference in growth rate could be detected between the treated cells and the control. MI and multiplication of the populations of cells given light alone or HPD alone did not differ from the control. Populations treated with combinations of HPD and light contained higher numbers

TABLE I.—*Mean colony diameter for NHIK 3025 cells exposed to 0·25 mg/ml HPD in medium with 30% serum. The cells were exposed to light in different phases in the cell cycle and incubated for 7 days in 37°C*

| Light exposure (min) | Time between synchronization and light exposure (phase) | Surviving fraction ± s.e. | Mean colony diameter ± s.e. (mm) |
|---|---|---|---|
| 0 | — | 1·00 | 0·76 ± 0·03 |
| 2 | 2 h ($G_1$) | 0·89 ± 0·06 | 0·73 ± 0·04 |
| 2 | 10 h (S) | 0·57 ± 0·10 | 0·64 ± 0·03 |
| 2 | 18 h ($G_2$, M, $G_1$) | 0·75 ± 0·05 | 0·66 ± 0·03 |
| 4 | 2 h ($G_1$) | 0·77 ± 0·10 | 0·60 ± 0·03 |
| 4 | 10 h (S) | 0·11 ± 0·03 | 0·46 ± 0·03 |
| 4 | 18 h ($G_2$, M, $G_1$) | 0·30 ± 0·05 | 0·49 ± 0·03 |

FIG. 1.—Multiplication of asynchronous cells treated with HPD (0·25 mg/ml in medium with 30% serum) and light, 4 h after subculture. The upper part shows increase in the multiplicity of microcolonies and the lower part shows MI for the same cell populations. Symbols: ●, neither HPD nor light; ×, 2 min light and no HPD; ○, HPD and no light; ▲, HPD and 1 min light; △, HPD and 2 min light.

FIG. 2.—Entrance into and passage through mitosis for synchronized cells treated with 0·25 mg/ml HPD and 2 min light in different stages of the cell cycle (arrows). Symbols: ●, control; ○, cells exposed to light in $G_1$; △, cells exposed to light in S; ▲, cells exposed to light in $S/G_2$.

of mitoses for a period after light treatment. It can be seen that the number of mitoses was abnormally high until normal multiplication of the treated cell populations was resumed. A study of stained chromosomes in cells from populations of HPD and light-treated cells indicated that the number of cells in metaphase was increased.

Essentially similar results (data not shown) were obtained when cells were treated in the absence of serum. Furthermore, HP (free base, Sigma) in medium with 30% serum combined with light influenced the cell proliferation as above.

*Multiplication of synchronized cells*

Synchronized cells exposed to HPD and light were allowed to form colonies at 37°C for 7 days. The mean colony diameter was measured with an ocular micrometer (Table I).

The mean diameter of colonies arising from surviving cells was less than in the control cells. The sensitivity of the cells was greater in S than in $G_1$, both in terms of cell inactivation and growth delay.

Synchronized cells given HPD and light in $G_1$, S and $S/G_2$ traverse the cell cycle as shown in Fig. 2. It can be seen that the prolongation of interphase is small compared to the prolongation of mitosis. MI reaches a high value for all cell populations, though not as high for cells treated in $G_1$ as for cells treated later in the cell cycle.

Multiplication of cells selected in mitosis after treatment with HPD and 2 min light

FIG. 3.—Mutliplication of cells selected by mitotic shake-off. Solid symbols show the multiplication of control cells. Open symbols show the multiplication of cells given 0·25 mg/ml HPD and 2 min light before selection. (Time between treatment and selection: ○, 4½ h; □, 8½ h.)

in interphase is shown in Fig. 3. Control cells completed mitosis by 1 h after mitotic selection, whereas only ~ 50% of the cells treated with HPD and light divided during 5 h after mitotic selection. Mean doubling time of the cell population treated with HPD and light was 20 h at > 25 h after mitotic selection (Fig. 3) compared to 19 h for the control cell population.

Multiplication of HPD-labelled cells exposed to light while in mitosis was tested

FIG. 4.—Passage out of mitosis for cells given 0·25 mg/ml HPD and light immediately before synchronization. The percentage of the cells that are still in mitosis at different times after synchronization is shown. Length of light doses (min): ●, 0; ▲, 1; △, 2; ○, 4.

in the following way. Mitotic selection was performed as usual (see above). Preceding the selection, cells were given a period of 30 min in contact with HPD, followed by light exposure and medium change. As soon as possible after light exposure, the flasks were placed on the reciprocal shaker and mitotic selection was performed. Thus, by observing the selected mitoses, the effect of treatment with HPD and light upon the entrance into interphase could be assayed (Fig. 4). About 50% of the cells given 0, 1 or 2 min light went out of mitosis during the first 30 min after selection. After that time entrance into interphase was slower for the cells given light. Cells given 4 min light proceeded slower than the untreated cells at all times after synchronization. This means that cells given light in the later stages of the mitosis entered interphase at the same rate as the control. The slower entrance in interphase after 30 min must reflect the properties of cells treated early in the mitosis.

The same cell populations (*i.e.* treated with HPD and 1 or 2 min light in mitosis) were followed until they reached the next mitosis. These populations behaved like the cell population given treatment in $G_1$ (see Fig. 2) with respect to prolongation of interphase and MI (data not shown).

Plating efficiency of selected mitosis was lower for cells treated with HPD and light than in the control series (Table II). The results indicate that only about half

TABLE II.—*Plating efficiency (PE) of selected mitosis. Asynchronous cells were treated with 0·25 mg/ml HPD in medium with 30% serum and light before selection*

| Experiment No. | Light exposure (min) | Time between HPD + light and selection (h) | PE (%) |
|---|---|---|---|
| 1 | 0 | 5½ | 87·5 |
| 1 | 2 | 5½ | 39·4 |
| 1 | 2 | 7½ | 37·8 |
| 2 | 0 | 15 | 76·5 |
| 2 | 2 | 15 | 26·5 |
| 3 | 2·5 | 24 | 35·7 |

of the cells subjected to prolonged mitosis survive.

### DISCUSSION

The dose-dependent growth-delay demonstrated herein is mainly due to inhibition in the first cell cycle after treatment with porphyrins and light. The increase in the number of cells in mitotis after treatment, seen in Fig. 1, confirms the earlier indications of mitotic inhibition (Christensen & Moan, 1980*b*) and an increased fraction of cells with DNA content characteristic of $G_2$ and mitosis (Gomer, 1980). Although mitosis is the stage which is mainly influenced by the treatment, a small prolongation of interphase has also been observed (Fig. 2).

Judged from the data in Figs 1, 4 and Table I, it is unlikely that the inhibition of cell growth by photoactivated porphyrins lasts longer than one cell generation. In the experiment shown in Fig. 1, the cells given 2 min light suffered from a delay of about 14 h in doubling of the multiplicity. The corresponding delay after many generations can be calculated from the colony diameters of treated and untreated cells (Table I). A typical reduction of the colony area is 40% (0·45 to 0·32 $mm^2$, corresponding to the reduction in diameter from 0·76 to 0·65 mm). Assuming that the area of a colony is proportional to the cell number and that the cell number increases exponentially, a delay of only 9 h can be estimated. This delay is less than the delay caused by inhibition in the progression through the first mitosis (Fig. 1). One should, however, not assume that cell number increases exponentially in colonies independently of their size. It is expected that the multiplication of cells in a colony will be inhibited as the cell number increases. Contact inhibition in the colonies arising from untreated cells may be the reason for the relatively small delay in multiplication found when colony area is used as a parameter. The same phenomenon may explain why our results conflict with the previous finding that cells treated with HP and light formed colonies of the same size as the control cells (Moan *et al.*, 1979). In that study the cells were incubated longer than ours, and the mean area of the colonies was more than twice that found in this study. The observation by Gomer & Smith (1980) of reduction in colony size is in accordance with the present findings.

The data in Fig. 3 indicate that cells treated during interphase may be delayed several hours in completing mitosis. Fig. 4 shows that the same is true for about half the cells given HPD and 1 or 2 min light during mitosis. The rest of the cells completed mitosis at the same rate as the control cells. This fact, in conjunction with the increased number of cells in metaphase in treated cell populations, indicates that the exposure to HPD and light induces a block in the progression through metaphase or in the transition between metaphase and anaphase.

Microscopic observation of cells arrested in mitosis indicates that some of the cells undergo lysis. The direct measurements of viability of selected mitoses also show that some of the treated cells are unable to survive the first mitosis after treatment. Although it can be argued that the selection *per se* and cooling of mitosis may fix potentially lethal damage (Pettersen *et al.*, 1977), it is probable that the lower survival of treated cells also reflects an irreversible inhibition in mitosis. The method of selection induces little damage to the cells, as shown by the fact that both untreated cells selected in mitosis and trypsinized cells have plating efficiencies of 75–90%.

Thus it is probable that two modes of cell death are participating in porphyrin-sensitized photodynamic inactivation of cells: on the one hand, cells are lysed shortly after irradiation (Moan *et al.*, 1979) and on the other, some cells are damaged in a way that makes them unable to complete the next mitosis. The mechanisms behind these modes of cell inactivation are unclear. Most probably, the acute lysis of cells after irradiation is caused by impaired membrane permeability (Kessel, 1977; Moan & Christensen, 1979). The delay in traverse through the first cell cycle and mitotic inhibition may be due to a variety of cellular functions that have been shown to be damaged by porphyrins and light. In relation to multiplication, protein synthesis is important. Rønning *et al.* (1979) have shown that a doubling of the amount of protein is required for the traverse through one cell cycle for NHIK 3025 cells. Protein synthesis is inhibited by porphyrins combined with light (Wakulchik *et al.*, 1980) and this inhibition may be one of the reasons for the observed effects upon the duration of interphase and mitosis. Mitosis itself is a complicated process involving most organelles in the cell. If the indication of a block in metaphase is correct, it is probable that the synthesis or polymerization of the proteins forming the mitotic spindle is inhibited. The small amount of damage to cellular DNA by photoactivated porphyrins that has been found (Gomer *et al.*, 1978; Moan *et al.*, 1980) may also have some consequences for cell proliferation.

These results may have implications for the clinical use of porphyrins and light in cancer therapy. It is of general importance to know the effect of a mode of therapy on the cell kinetics in the tumour. Furthermore, if the results found here for cells *in vitro* can be extrapolated to the *in vivo* situation, one would expect to find a large fraction of the tumour-cell population in mitosis at a certain time after light irradiation. This may be taken advantage of in combination therapy in the future.

This study was supported by the Norwegian Cancer Society (Landsforeningen mot Kreft) and The Norwegian Research Council for Science and the Humanities. The author is indebted to Dr Johan Moan for constructive discussions.

### REFERENCES

Christensen, T. & Moan, J. (1980*a*) Pyotodynamic effect of hematoporphyrin (HP) on cells cultivated *in vitro*. In *Lasers in Photomedicine and Photobiology*. Ed. Pratesi & Sacci. Berlin: Springer. p. 87.

Christensen, T. & Moan, J. (1980*b*) Effect of hematoporphyrin and light on cell multiplication *in vitro*. *8th Int. Cong. Photobiol.*, p. 299.

Christensen, T., Moan, J., Wibe, E. & Oftebro, R. (1979) Photodynamic effect of haematoporphyrin throughout the cell cycle of the human cell line NHIK 3025 cultivated *in vitro*. *Br. J. Cancer*, **34**, 64.

Dougherty, T. J., Kaufman, J. E., Goldfarb, A., Weisshaupt, K. R., Boyle, D. & Mittleman, A. (1978) Photoradiation therapy for the treatment of malignant tumours. *Cancer Res.*, **38**, 2628.

Gomer, C. J., Weishaupt, K. R. & Dougherty, T. J. (1978) The effects of hematoporphyrin derivative and visible red light on V-79 cells. *Radiat. Res.*, **74**, 586.

Gomer, C. J. (1980) Cellular studies pertaining to hematoporphyrin photoradiation therapy. *Radiat. Res.*, **83**, 374.

Gomer, C. J. & Smith, D. M. (1980) Photoinactivation of Chinese hamster cells by hematoporphyrin derivative and red light. *Photochem. Photobiol.*, **32**, 341.

Kelly, J. F. & Snell, M. E. (1976) Hematoporphyrin derivative: A possible aid in the diagnosis and therapy of carcinoma of the bladder. *J. Urol.*, **115**, 150.

Kessel, D. (1977) Effects of photoactivated porphyrins at the cell surface of leukemia L 1210 cells. *Biochemistry*, **16**, 3443.

Lipson, R., Blades, E. & Olsen, A. (1961) The use of a derivative of hematoporphyrin in tumor detection. *J. Natl Cancer Inst.*, **26**, 1.

Moan, J. & Christensen, T. (1979) Photodynamic inactivation of cancer cells *in vitro*. Effect of irradiation temperature and dose fractionation. *Cancer Lett.*, **6**, 331.

Moan, J., Pettersen, E. O. & Christensen, T. (1979) The mechanism of photodynamic inactivation of human cells *in vitro* in the presence of haematoporphyrin. *Br. J. Cancer*, **39**, 398.

Moan, J., Waksvik, H. & Christensen, T. (1980) DNA single strand breaks and sister chromatid exchanges induced by treatment with hematoporphyrin and light or by X-rays in human cells of the line NHIK 3025. *Cancer Res.*, **40**, 2915.

Nordbye, K. & Oftebro, R. (1969) Establishments of four new cell strains from human uterine cervix: I. *Exp. Cell Res.*, **58**, 458.

Oftebro, R. & Nordbye, K. (1969) Establishments of four new cell strains from human uterine cervix: II. *Exp. Cell Res.*, **58**, 459.

Pettersen, E. O., Bakke, O., Lindmo, T. & Oftebro, R. (1977) Cell cycle characteristics of synchronized and asynchronous populations of human cells and effect of cooling of selected mitotic cells. *Cell Tissue Kinet.*, **10**, 511.

Pettersen, E. O., Oftebro, R. & Brustad, T. (1973) X-ray inactivation of human cells in tissue culture under aerobic and extremely hypoxic conditions in the presence and absence of TMPN. *Int. J. Radiat. Biol.*, **24**, 285.

Puck, T. T., Cieciura, S. J. & Fisher, H. (1957) Clonal growth *in vitro* of human cells with fibroblastic morphology. *J. Exp. Med.*, **106**, 145.

Rønning, Ø. W., Pettersen, E. O. & Seglen, P. O. (1979) Protein synthesis and protein degradation through the cell cycle of human NHIK 3025 cells *in vitro*. *Exp. Cell Res.*, **123**, 63.

Schothorst, A. A., De Haas, C. A. C. & Suurmond,

D. (1980) Photochemical damage to skin fibroblasts caused by protoporphyrin and violet light. *Arch. Dermatol. Res.*, **268**, 31.

WAKULCHIK, S. D., SCHILTZ, J. R. & BICKERS, D. R. (1980) Photolysis of protoporphyrin-treated human fibroblasts *in vitro*: Studies on the mechanism. *J. Lab. Clin. Med.*, **96**, 158.

WEISHAUPT, K. R., BOYLE, D. G., MENSINGER, M. & DOUGHERTY, T. J. (1979) Cellular and tissue repair of photodynamic damage. *Proc. Am. Ass. Cancer Res.*, **20**, 918.

WIBE, E., OFTEBRO, R., CHRISTENSEN, T., LALAND, S. G., PETTERSEN, E. O. & LINDMO, T. (1978) Inhibitory effects of the new mitotic inhibitor 5-chloropyrimidin-2-one and of vincristine on human cells *in vitro*. *Cancer Res.*, **38**, 560.